# Economic burden of hepatitis B infection among patients with diabetes

**DOI:** 10.1080/21645515.2015.1127488

**Published:** 2016-04-06

**Authors:** Gaurav Deshpande, Andrew J. Klink, Rahul Shenolikar, Joseph Singer, Debra F. Eisenberg Lawrence, Girishanthy Krishnarajah

**Affiliations:** a HealthCore, Inc., Wilmington, DE, USA; b GSK Vaccines, Research Triangle Park, NC, USA; c GSK Vaccines, Philadelphia, PA, USA

**Keywords:** diabetes, financial burden, hepatitis B, healthcare utilization, costs

## Abstract

Despite ACIP recommendation and cost-effectiveness established in those 19–59 y old diabetes patients the uptake of Hepatitis B vaccine in diabetes patients is low. There is need to highlight the impact of Hepatitis B virus (HBV) infection in diabetes patients in terms of healthcare utilization and costs to recognize the burden of HBV in this population.

This retrospective claims analysis included patients with diabetes and HBV (cases; n=1,236) and those with diabetes without HBV (controls; n=4,944), identified by ICD-9-CM diagnosis codes. Cases were matched with 4 controls using propensity score matching. Healthcare utilization and cost were compared; incremental effect of HBV infection was assessed using multivariate analysis.

In the adjusted analyses, the mean number of hospitalizations (0.6 vs 0.4), outpatient service visits (34.2 vs. 20.4), and office visits (10.9 vs. 9.8) were 41%, 68%, and 11% higher, respectively, in cases vs. controls (all p<0.05). Gastroenterologist visits (0.8 vs. 0.2) and infectious disease visits (0.1 vs. 0.0) were 80% and 18% higher in subset of case and controls with these events. Cases ($39,435) incurred $16,397 incremental total costs compared with controls ($23,038). Medical ($30,968 vs. $17,765) and pharmacy costs ($8,029 vs. $5,114) were both significantly higher for cases (p < 0.0001).

Healthcare utilization and costs were higher among patients with diabetes and HBV than in those with diabetes alone. These results provide evidence supporting the need for HBV vaccination among unvaccinated diabetes patients.

## ABBREVIATIONS

ACIP: Advisory Committee on Immunization Practices
aDCSI: adapted Diabetes Comorbidity Severity Index
ED: emergency department
HBV: hepatitis B virus
HIRD^SM^: HealthCore Integrated Research Database
ICD-9-CM: *International Classification of Diseases, Ninth Revision, Clinical Modification*
NQF: National Quality Forum; SD, standard deviation

## Introduction

As a risk for people with diabetes mellitus, hepatitis B virus (HBV) infection is under-recognized. Adults with diabetes have a 60% higher prevalence rate of HBV infection^1^ and a higher case-fatality rate than those without diabetes.^2^ Rates of chronic liver disease and hepatocellular carcinoma are also higher in people with diabetes.^3^ The annual incidence of reported cases of HBV infection among adults with diabetes is 1.8 per 100,000,^2^ which is likely an underestimate when asymptomatic infection, underdiagnosis, and under-reporting are considered.^4^ The increased risk of HBV infection in adults with diabetes holds for both genders, across ethnic and racial groups, and for those without traditional HBV risk behaviors, such as use of injected drugs or multiple sexual partners.^1^

HBV is stable and remains viable on surfaces up to a week,^5,6^ making the virus transmissible through contaminated equipment used for routine diabetes care and blood glucose monitoring.^1,2,4^ Between 1995 and 2006, 86% of the HBV outbreaks in long-term care facilities occurred among patients with diabetes who received assisted blood glucose monitoring.^7^ People with diabetes can be exposed to HBV infection outside of institutional settings, such as physician offices, hospitals, health fairs, and schools, if assisted glucose monitoring is offered.^1^

After reviewing the HBV-related morbidity and mortality and the limitations of infection control measures, the Advisory Committee on Immunization Practices (ACIP) recommended in 2011 that all previously unvaccinated adults aged 19 through 59 y with diabetes mellitus be vaccinated against hepatitis B as soon as possible after a diagnosis of diabetes.^8^ In 2013, vaccination coverage for persons with diabetes was 26.3% for those aged 19–59 y and 13.9% for those aged ≥60 years.^9^

Although cost analyses have been conducted for HBV treatment in USA^10-13^ and for diabetes,^14-18^ as well as for the cost-effectiveness of HBV vaccination in adults with diabetes,^4^ research is lacking on the impact of both HBV in diabetes patients in terms of healthcare utilization and costs. Such research can help highlight the need to vaccinate diabetes patients eligible for vaccination. To fill this research gap, the primary objective of the current study was to measure healthcare utilization and costs for patients with both HBV infection and diabetes compared with patients with diabetes alone using a real-world population of adults enrolled in large commercial health plans.

## Results

### Patient characteristics

**Figure 1.**
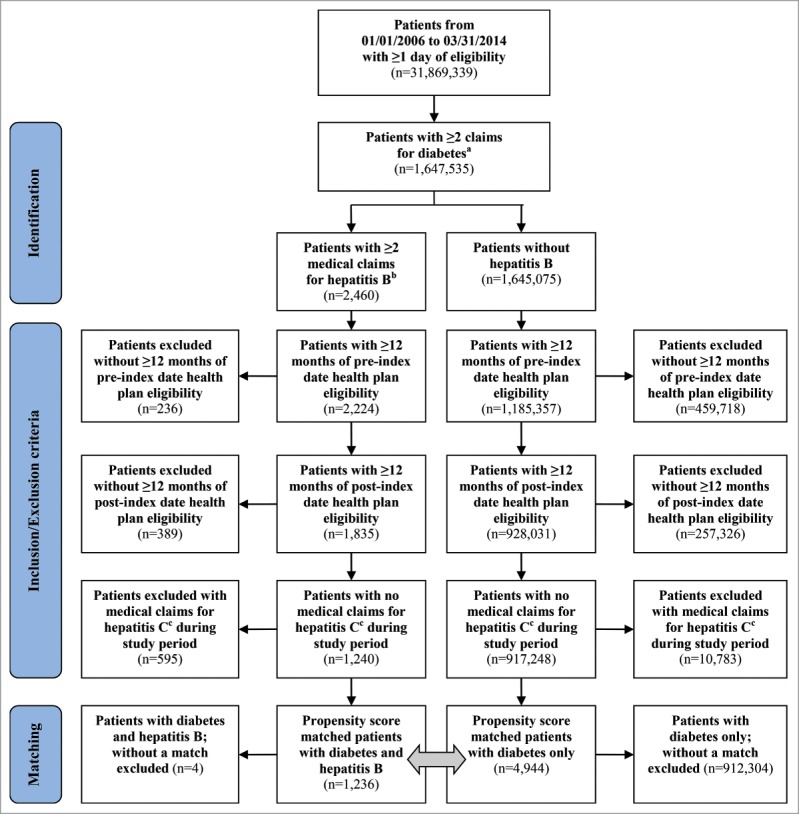
Patient Attrition. ^a^At least 2 medical claims for diabetes at least 30 d apart (250.xx); OR at least 1 medical claim for diabetes and at least 1 pharmacy claim for a diabetes medication (GPI 27xx or 39100016x). ^b^Hepatitis B identified by ICD-9-CM codes 070.2x or 070.3x. ^c^Hepatitis C identified by ICD-9-CM codes 070.44, 070.54, 070.70, 070.71, 070.41, 070.51, or V02.62.

A total of 918,488 patients (1,240 patients with diabetes and HBV infection [cases]; 917,248 patients with diabetes but no HBV infection [controls]) met all inclusion criteria for the study (Fig. 1). After propensity score matching, the final study population was 6,180 patients (1,236 cases; 4,944 controls). Prior to matching, the 2 cohorts were statistically different on a number of categories, including age, gender, severity of diabetes, and comorbidities (Table 1). After matching, the two groups were similar in nearly all baseline covariates.

**Table 1.** Baseline Patient Clinical Characteristics Included in Propensity Score by Matched Status.^a^

**Clinical Characteristics**	**Prior to matching (N=918,488)**			**Matched cohort (N=6,180)**		
	**Diabetes without HBV (n = 917,248)**	**Diabetes with HBV (n = 1,240)**	**p-value**^b^	**Diabetes without HBV (n = 4,944)**	**Diabetes with HBV (n = 1,236)**	**p-value**^b^
Age (on index date), mean, median (SD)	56.6, 57.0 (14.33)	54.0, 54.0 (11.36)	<0.0001	54.1, 55.0 (14.15)	54.0, 54.0 (11.36)	0.8164
Gender, n (%)						
Female	430,681 (47.0)	433 (34.9)	<0.0001	1,742 (35.2)	432 (35.0)	0.8521
Geographic region of healthplan, n (%)			<0.0001			0.9218
Northeast	177,957 (19.4)	290 (23.4)		1,113 (22.5)	290 (23.5)	
Midwest	292,693 (31.9)	164 (13.2)		651 (13.2)	164 (13.3)	
South	252,570 (27.5)	197 (15.9)		776 (15.7)	196 (15.9)	
West	147,377 (16.1)	557 (44.9)		2,261 (45.7)	554 (44.8)	
Unknown	46,651 (5.1)	32 (2.6)		143 (2.9)	32 (2.6)	
Index year, n (%)			<0.0001			0.9608
2007	407,886 (44.5)	405 (32.7)		1,599 (32.3)	405 (32.8)	
2008	120,931 (13.2)	183 (14.8)		696 (14.1)	183 (14.8)	
2009	106,874 (11.7)	179 (14.4)		760 (15.4)	178 (14.4)	
2010	87,960 (9.6)	164 (13.2)		664 (13.4)	164 (13.3)	
2011	92,531 (10.1)	169 (13.6)		678 (13.7)	167 (13.5)	
2012	79,581 (8.7)	121 (9.8)		461 (9.3)	120 (9.7)	
2013	21,485 (2.3)	19 (1.5)		86 (1.7)	19 (1.5)	
Length of pre-index eligibility (months), mean, median (SD)	19.6, 13.3 (13.56)	25.8, 18.5 (16.60)	<0.0001	25.8, 14.5 (19.43)	25.7, 18.4 (16.54)	0.8379
Presence of diabetes during pre-index period, n (%)	624,248 (68.1)	860 (69.4)	0.3272	3,368 (68.1)	857 (69.3)	0.4119
Severity of diabetes^c^, n (%)						
aDCSI score, mean, median (SD)	0.9, 0.0 (1.52)	1.1, 0.0 (1.91)	<0.0001	1.2, 0.0 (1.77)	1.1, 0.0 (1.90)	0.6690
Mild (aDCSI=0)	558,858 (60.9)	747 (60.2)		2,812 (56.9)	745 (60.3)	
Moderate (aDCSI=1-4)	321,362 (35.0)	398 (32.1)		1,823 (36.9)	398 (32.2)	
1	132,771 (41.3)	148 (37.2)		589 (32.3)	148 (37.2)	
2	109,483 (34.1)	141 (35.4)		704 (38.6)	141 (35.4)	
3	45,218 (14.1)	49 (12.3)		280 (15.4)	49 (12.3)	
4	33,890 (10.5)	60 (15.1)		250 (13.7)	60 (15.1)	
Severe (aDCSI=5-13)	37,028 (4.0)	95 (7.7)		309 (6.3)	93 (7.5)	
Comorbidities, n (%)						
HIV	1,233 (0.1)	36 (2.9)	<0.0001	100 (2.0)	33 (2.7)	0.1608
Hypertension	572,813 (62.4)	677 (54.6)	<0.0001	2,701 (54.6)	674 (54.5)	0.9491
Hyperlipidemia	544,942 (59.4)	693 (55.9)	0.0116	2,791 (56.5)	691 (55.9)	0.7292
Coronary artery disease	155,949 (17.0)	210 (16.9)	0.9504	829 (16.8)	209 (16.9)	0.9052
Congestive heart failure	50,751 (5.5)	105 (8.5)	<0.0001	391 (7.9)	103 (8.3)	0.6224
Peripheral vascular disease	41,409 (4.5)	47 (3.8)	0.2196	180 (3.6)	47 (3.8)	0.7868
Cerebrovascular disease	62,410 (6.8)	79 (6.4)	0.5450	343 (6.9)	79 (6.4)	0.4960
Dementia	4,212 (0.5)	5 (0.4)	0.7708	23 (0.5)	5 (0.4)	0.7763
Chronic obstructive pulmonary disease	125,224 (13.7)	134 (10.8)	0.0035	547 (11.1)	134 (10.8)	0.8232
Rheumatological disease	17,222 (1.9)	28 (2.3)	0.3240	126 (2.5)	28 (2.3)	0.5678
Peptic ulcer disease	8,616 (0.9)	35 (2.8)	<0.0001	132 (2.7)	35 (2.8)	0.7537
Hemiplegia or paraplegia	3,236 (0.4)	5 (0.4)	0.7647	26 (0.5)	5 (0.4)	0.5891
Moderate or severe renal disease	43,704 (4.8)	170 (13.7)	<0.0001	702 (14.2)	168 (13.6)	0.5833
Malignancy	58,841 (6.4)	104 (8.4)	0.0046	445 (9.0)	104 (8.4)	0.5168
Metastatic solid tumor	5,972 (0.7)	17 (1.4)	0.0017	83 (1.7)	17 (1.4)	0.4496
AIDS	1,233 (0.1)	36 (2.9)	<0.0001	100 (2.0)	33 (2.7)	0.1608
Other liver disease	33,379 (3.6)	367 (29.6)	<0.0001	1,485 (30.0)	363 (29.4)	0.6466
Healthcare utilization in pre-index period^d^, mean, median (SD)						
Inpatient hospitalizations	0.2, 0.0 (0.70)	0.3, 0.0 (0.87)	0.0744	0.3, 0.0 (0.83)	0.3, 0.0 (0.86)	0.4500
Office visits	7.6, 6.0 (7.78)	8.0, 5.0 (8.47)	0.1379	8.0, 6.0 (8.06)	8.0, 5.0 (8.45)	0.9863

aDCSI=adapted Diabetes Comorbidity Severity Index; HBV=hepatitis B virus; SD=standard deviation

a Baseline period includes the 12 months pre-index for each patient

b p-value calculated using *t*-test for continuous variables and χ^2^ test for categorical variables, comparing diabetes without hepatitis B to all diabetes with hepatitis B.

c Severity of diabetes as calculated by the adapted Diabetes Comorbidity Severity Index (aDCSI)

d Reported for healthcare utilization in the pre-index period where it is not related to hepatitis B or its related complications (ie, cirrhosis, decompensated cirrhosis, liver cancer, fulminant hepatic failure, or liver transplant)

Overall, patients in the matched cohorts had a mean age of 54 y and the majority was men. The majority of patients in both cohorts (60.3% cases and 56.9% controls) had mild diabetes; 32.2% of cases and 36.9% of controls had moderate diabetes. The most common comorbidities at baseline in cases and controls were hyperlipidemia (55.9% vs. 56.5%), hypertension (54.5% vs. 54.6%), and other liver disease (29.4% vs. 30.0%). Among patients with both diabetes and HBV infection, 282 patients (22.8%) had late-stage liver disease identified during the post-index period.

### Healthcare utilization

Patients with diabetes plus HBV infection had greater healthcare resource utilization than patients with diabetes alone (Table 2). The mean adjusted number of hospitalizations [0.6 (95% CI 0.5–0.7) vs 0.4 (95% CI 0.4–0.5); p < 0.0001], office visits [10.9 (95% CI 10.4–11.4) vs 9.8 (95% CI 9.6–10.1); p < 0.0001], gastroenterologist visits [0.8 (95% CI 0.7–0.9) vs 0.2 (95% CI 0.2–0.2); p < 0.0001], infectious disease specialist visits [0.1 (95% CI 0.1–0.1) vs 0.0 (95% CI 0.0–0.1); p = 0.0001], and outpatient visits [34.2 (95% CI 32.0–36.5) vs 20.4 (95% CI 19.5–21.3); p < 0.0001] was higher for cases than controls. The number of ED visits was similar between the two groups [0.3 for both cases (95% CI 0.2–0.3) and controls (95% CI 0.3–0.4); p = 0.0124]. A similar pattern was observed among patients with at least one visit, with utilization higher for cases than controls for the mean adjusted number of hospitalizations [1.5 (95% CI 1.3–1.6 vs 1.0 (95% CI 1.0–1.1); p < 0.0001] and gastroenterologist visits [1.6 (95% CI 1.5–1.8) vs 0.9 (95% CI 0.8–1.0); p < 0.0001]. Infectious disease specialist visits [1.7 (95% CI 1.3–2.2) vs 1.4 (95% CI 1.2–1.8); p = 0.2527] and ED visits were similar between the two groups [0.8 visits for both cases (95% CI 0.7–0.9) and controls (95% CI 0.8–0.9); p=0.7478].

**Table 2.** Multivariate Analysis of Annualized Healthcare Utilization^a^.^a^

**Place of Service**	**Diabetes without HBV (n=4,944)**	**Diabetes with HBV (n=1,236)**	**95% CI p-value**^b^	**Diabetes without HBV Adjusted Mean**^c,d^^c,d^**(95% CI)**	**Diabetes with HBV Adjusted Mean**^c,d^^c,d^**(95% CI)**	**IRR**^c^**(95% CI) p-value**^b^
**Inpatient hospitalizations**						
Patients with ≥1 hospitalization, n (%)	1,879 (38.0)	454 (36.7)	0.84–1.09 0.5205			
Number of hospitalizations among all patients, mean, median (SD)	0.4, 0.0 (0.87)	0.5, 0.0 (1.24)		0.4 (0.4–0.5)	0.6 (0.5–0.7)	1.41 (1.26–1.58) <0.0001
Number of hospitalizations among patients with ≥1 hospitalization, mean, median (SD)	1.0, 0.6 (1.19)	1.4, 0.8 (1.71)		1.0 (1.0–1.1)	1.5 (1.3–1.6)	1.43 (1.29–1.58) <0.0001
LOS among all patients, mean, median (SD)	2.2, 0.0 (7.80)	3.6, 0.0 (13.14)		2.5 (2.2–2.7)	4.0 (3.4–4.7)	1.61 (1.36–1.90) <0.0001
LOS among patients with ≥1 hospitalization, mean, median (SD)	5.7, 1.6 (11.83)	9.7, 2.7 (20.29)		5.9 (5.4–6.4)	9.8 (8.6–11.3)	1.67 (1.45–1.92) <0.0001
**ED visits**						
Patients with ≥1 visit, n (%)	1,900 (38.4)	404 (32.7)	0.69–0.90 0.0003			
Number of visits among all patients, mean (SD)	0.3, 0.0 (1.15)	0.2, 0.0 (0.57)		0.3 (0.3–0.4)	0.3 (0.2–0.3)	0.84 (0.74–0.96) 0.0124
Number of visits among patients ≥1 visit, mean (SD)	0.8, 0.5 (1.76)	0.8, 0.5 (0.77)		0.8 (0.8–0.9)	0.8 (0.7–0.9)	0.98 (0.86–1.12) 0.7478
**Office visits**						
All office visits						
Patients with ≥1 visit, n (%)	4,911 (99.3)	1,234 (99.8)	0.95–17.67 0.0579			
Number of visits among all patients, mean (SD)	9.1, 6.7 (7.96)	10.0, 7.5 (8.42)		9.8 (9.6–10.1)	10.9 (10.4–11.4)	1.11 (1.06–1.16) <0.0001
Number of visits among patients with ≥1 visit, mean (SD)	9.1, 6.8 (7.95)	10.0, 7.6 (8.42)		9.9 (9.6–10.2)	10.9 (10.4–11.6)	1.10 (1.05–1.15) <0.0001
Visits to a gastroenterologist						
Patients with ≥1 visit, n (%)	935 (18.9)	615 (49.8)	3.72–4.88 <0.0001			
Number of visits among all patients, mean (SD)	0.2, 0.0 (0.61)	0.8, 0.0 (1.31)		0.2 (0.2–0.2)	0.8 (0.7–0.9)	4.73 (4.20–5.33) <0.0001
Number of visits among patients with ≥1 visit, mean (SD)	0.9, 0.5 (1.15)	1.6, 1.1 (1.49)		0.9 (0.8–1.0)	1.6 (1.5–1.8)	1.80 (1.62–1.99) <0.0001
Visits to an infectious disease specialist						
Patients with ≥1 visit, n (%)	137 (2.8)	61 (4.9)	1.31–2.45 0.0003			
Number of visits among all patients, mean (SD)	0.0, 0.0 (0.35)	0.1, 0.0 (0.53)		0.0 (0.0–0.1)	0.1 (0.1–0.1)	2.13 (1.44–3.15) 0.0001
Number of visits among patients with ≥1 visit, mean (SD)	1.4, 0.8 (1.53)	1.7, 1.0 (1.70)		1.4 (1.2–1.8)	1.7 (1.3–2.2)	1.18 (0.89–1.57) 0.2527
**Outpatient services**^e^						
Patients with ≥1 visit, n (%)	4,887 (98.8)	1,232 (99.7)	1.26–9.63 0.0159			
Number of visits among all patients, mean (SD)	17.7, 9.3 (30.61)	30.5, 11.3 (53.88)		20.4 (19.5–21.3)	34.2 (32.0–36.5)	1.68 (1.57–1.79) <0.0001
Number of visits among patients with ≥1 visit, mean (SD)	17.9, 9.4 (30.73)	30.6, 11.4 (53.94)		20.6 (19.7–21.5)	34.2 (32.0–36.5)	1.66 (1.56–1.78) <0.0001
**Skilled nursing facility services**						
Patients with ≥1 visit, n (%)	561 (11.3)	140 (11.3)	0.83–1.24 0.8606			
**Pharmacy prescriptions**						
Patients with ≥1 pharmacy claim, n (%)	4,703 (95.1)	1,179 (95.4)	0.80–1.53 0.5326			
Number of pharmacy claims among all patients, mean (SD)	47.4, 38.5 (39.72)	41.5, 30.4 (39.76)		58.9 (56.7–61.2)	51.2 (48.2–54.3)	0.87 (0.82–0.92) <0.0001
Number of pharmacy claims among patients with ≥1 pharmacy claim, mean (SD)	49.9, 41.1 (39.21)	43.6, 32.4 (39.62)		60.2 (58.3–62.2)	52.2 (49.6–55.0)	0.87 (0.82–0.91) <0.0001
Number of unique medication classes among all patients, mean (SD)	6.8, 5.5 (5.42)	6.9, 5.0 (6.26)		7.7 (7.5–7.9)	7.7 (7.3–8.0)	1.00 (0.95–1.05) 0.9474
Number of unique medication classes among patients with ≥1 pharmacy claim, mean (SD)	7.2, 5.8 (5.35)	7.2, 5.2 (6.26)		7.9 (7.7–8.1)	7.8 (7.5–8.2)	1.00 (0.97–1.04) 0.8197

CI=confidence interval; ED=emergency department; HBV=hepatitis B virus; IRR=incidence rate ratio; LOS=length of stay; OR=odds ratio; SD=standard deviation

a Healthcare utilization was measured from the index date to the end of patients' follow up in the study and was annualized

b *p*-value was calculated using multivariate regression (ie, negative binomial regression for count variables and logistic regression for dichotomous variables) comparing patients with diabetes with HBV to patients with diabetes without HBV

c Patients with diabetes without HBV used as referent. Multivariate model adjusted for baseline insulin use and use of antidiabetic agents associated with hepatotoxicity

d Comparison of patients with diabetes and HBV to patients with diabetes without HBV; patients with diabetes without HBV used as referent

e Other outpatient services included, for example, laboratory procedures, etc.

### Healthcare costs

Mean adjusted total costs for cases ($39,435) were 71% higher compared with controls ($23,038). Total medical costs were $30,968 (95% CI $28,311-$33,874) in cases compared with $17,765 (95% CI $16,788-$18,802) in controls, and pharmacy costs were also higher for cases than controls (Table 3). Incremental costs were highest for outpatient services ($7,039) and inpatient hospitalizations ($6,008) and lowest for gastroenterologist ($67), infectious disease specialist ($8), and general office visits ($118). Costs for ED visits were lower for cases than controls, with incremental costs of $99. Among patients with at least one healthcare utilization event, costs were significantly higher for all healthcare utilization events except ED visits. There was no significant difference in costs in subset of patients that had at least one ED visit.

**Table 3.** Multivariate Analysis of Annualized Costs^a^.^a^

**Costs**	**Diabetes without HBV (n=4,944)**	**Diabetes with HBV (n=1,236)**	**DifferenceMean^b^ (%)**	**Diabetes without HBV Adjusted Mean**^c,d^^b,c^**(95% CI)**	**Diabetes with HBV Adjusted Mean**^c,d^^b,c^**(95% CI)**	**Incidence rate ratio**^c^	**95% CI**	***p-value***^d^
**Inpatient hospitalizations**								
All patients, mean, median (SD)	$7,604, $0 ($29,952)	$13,412, $0 ($59,513)	$5,808 (76.4)	$8,089 ($7,274–$8,994)	$14,097 ($11,877–$16,729)	1.74	1.47–2.06	<0.0001
Patients with ≥1 hospitalization, mean, median (SD)	$20,007, $7,266 ($45,968)	$36,514, $10,243 ($93,865)	$16,507 (82.5)	$19,341 ($17,860–$20,946)	$34,704 ($30,236–$39,831)	1.79	1.56–2.06	<0.0001
**ED visits**								
All patients, mean, median (SD)	$457, $0 ($1,423)	$365, $0 ($1,066)	−$92 (−20.1)	$504 ($461–$552)	$405 ($352–$466)	0.80	0.70–0.92	0.0020
Patients with ≥1 visit, mean, median (SD)	$1,189, $590 ($2,097)	$1,117, $642 ($1,626)	−$72 (−6.1)	$1,230 ($1,149–$1,315)	$1,157 ($1,029–$1,301)	0.94	0.84–1.06	0.3175
**Office visits**								
All patients, mean, median (SD)	$1,340, $791 ($3,427)	$1,451, $894 ($2,519)	$111 (8.3)	$1,421 ($1,367–$1,478)	$1,539 ($1,448–$1,636)	1.08	1.02–1.15	0.0106
Patients with ≥1 visit, mean, median (SD)	$1,349, $801 ($3,436)	$1,453, $896 ($2,520)	$104 (7.7)	$1,432 ($1,379–$1,488)	$1,543 ($1,454–$1,637)	1.08	1.02–1.14	0.0140
Visits to a gastroenterologist								
All patients, mean, median (SD)	$20, $0 ($78)	$89, $0 ($162)	$69 (345.0)	$21 ($19–$22)	$88 ($79–$99)	4.27	3.82–4.78	<0.0001
Patients with ≥1 visit, mean, median (SD)	$107, $59 ($152)	$179, $127 ($191)	$72 (67.3)	$108 ($100–$117)	$181 ($166–$198)	1.68	1.53–1.84	<0.0001
Visits to an infectious disease specialist								
All patients, mean, median (SD)	$5, $0 ($48)	$11, $0 ($82)	$6 (120.0)	$6 ($6–$7)	$14 ($13–$16)	2.23	2.01–2.46	<0.0001
Patients with ≥1 visit, mean, median (SD)	$167, $81 ($239)	$228, $130 ($300)	$61 (36.5)	$174 ($141–$215)	$240 ($179–$322)	1.38	1.02–1.86	0.0370
**Outpatient services**^e^								
All patients, mean, median (SD)	$6,238, $1,826 ($18,909)	$12,621, $2,286 ($35,678)	$6,383 (102.3)	$7,361 ($6,939–$7,807)	$14,400 ($13,137–$15,785)	1.96	1.78–2.14	<0.0001
Patients with ≥1 visit, mean, median (SD)	$6,311, $1,849 ($19,007)	$12,662, $2,293 ($35,728)	$6,351 (100.6)	$7,434 ($7,015–$7,878)	$14,423 ($13,179–$15,785)	1.94	1.77–2.12	<0.0001
**Skilled nursing facility services**								
All patients, mean, median (SD)	$310, $0 ($2,399)	$435, $0 ($3,006)	$125 (40.3)	$304 ($275–$336)	$481 ($408–$567)	1.58	1.35–1.86	<0.0001
Patients with ≥1 visit, mean, median (SD)	$2,730, $606 ($6,646)	$3,841, $632 ($8,191)	$1,111 (40.7)	$2,290 ($1,966–$2,666)	$3,406 ($2,599–$4,464)	1.49	1.12–1.98	0.0061
**Pharmacy prescriptions**								
All patients, mean, median (SD)	$3,918, $2,060 ($6,725)	$6,072, $2,672 ($9,624)	$2,154 (55.0)	$5,114 ($4,844–$5,400)	$8,029 ($7,369–$8,748)	1.57	1.44–1.71	<0.0001
Patients with ≥1 pharmacy claim, mean, median (SD)	$4,119, $2,270 ($6,835)	$6,365, $2,946 ($9,758)	$2,246 (54.5)	$5,233 ($4,989–$5,489)	$8,181 ($7,580–$8,831)	1.56	1.45–1.69	<0.0001
**Total medical costs**,^f^**mean, median (SD)**	$15,948, $4,145 ($42,226)	$28,284, $4,471 ($78,485)	$12,336 (77.4)	$17,765 ($16,788–$18,802)	$30,968 ($28,311–$33,874)	1.74	1.59–1.91	<0.0001
**Total costs**,^g^**mean, median (SD)**	$19,867, $7,373 ($44,010)	$34,356, $10,160 ($80,378)	$14,489 (72.9)	$23,038 ($21,921–$24,212)	$39,435 ($36,454–$42,655)	1.71	1.58–1.85	<0.0001

CI=confidence interval; ED=emergency department; HBV=hepatitis B virus; SD=standard deviation

a All-cause costs calculated as sum of plan-paid and patient-paid costs and were adjusted to 2014 Consumer Price Index information provided by the Bureau of Labor & Statistics. Costs were measured from index date to the end of patients' follow up in the study and were annualized

b Comparison between patients with diabetes with HBV to patients with diabetes without HBV; patients with diabetes without HBV used as referent

c Multivariate model adjusted for baseline insulin use and use of antidiabetic agents associated with hepatotoxicity

d *p*-value calculated using multivariate regression (ie, logistic regression with gamma transformation for cost variables) comparing patients with diabetes with HBV to patients with diabetes without HBV

e Other outpatient services included, for example, laboratory procedures, etc.

f Sum of inpatient, ED, office visit, and other outpatient visit costs

g Sum of total medical and pharmacy costs

### Impact of late-stage liver disease

Among cases (that is, patients with diabetes and who had HBV infection), utilization varied according to the specific type of late-stage liver disease identified. In a multivariate analysis, patients with diabetes and decompensated cirrhosis were more likely to be hospitalized and visit the ED than those without an HBV-associated liver disease (data not shown).

## Discussion

The results of this retrospective claims analysis demonstrated that HBV infection is associated with increased financial burden in patients with diabetes. Patients with diabetes plus HBV infection had higher healthcare utilization compared with those who had diabetes alone, in particular inpatient hospitalizations, office and specialist visits, and use of outpatient services. Patients with diabetes alone, however, were more likely to visit an ED than those with both diabetes and HBV infection, which is consistent with the greater number of office visits among patients with diabetes and HBV infection needed to manage their care. This finding did not hold when ED visits were compared among patients who had at least one ED visit. A possible explanation is that management of HBV infection in the outpatient setting resulted in fewer emergent care visits. Another explanation may be that patients with diabetes plus HBV may have been more likely to be admitted to the hospital, thus resulting in an underrepresentation of ED utilization among these patients. In fact, the mean number of hospitalizations was higher among patients with both diabetes and HBV infection, and their mean lengths of stay were 61% longer than those who had diabetes alone. The distribution for diseases unrelated to diabetes was not significantly different across cases and controls (as demonstrated in Table 1), but the costs in the two groups may still have differed and confounded the results. This study did not break down the costs that are disease related or not but the confounding due to differing costs may not be significant as the cases were matched to controls with a similar clinical profile (as shown in Table 1).

As expected, patients with diabetes and HBV infection also incurred higher annual medical and pharmacy costs compared with patients who had diabetes without HBV infection. Costs were also higher among the subset of patients with diabetes and HBV who had been diagnosed with late-stage liver disease during the follow-up period. These findings are consistent with previous research that demonstrated escalating costs associated with progressive liver disease among people with chronic HBV infection.^10^ Pharmacy costs were higher among patients with diabetes plus HBV than in those with diabetes alone despite higher pharmacy utilization among patients with diabetes alone. A possible explanation for this finding may be higher cost per medication for patients with diabetes plus HBV. Prior economic comparisons of HBV treatments in hypothetical populations concluded that cost-effectiveness varied widely depending on patient response rates and drug resistance.^11,13^ This study demonstrated higher costs among patients with diabetes and HBV in a real world environment using administrative claims.

A strength of this study was the large, geographically diverse population, and the ability to examine actual healthcare use and costs. However, the study had limitations. The data were extracted from administrative claims, which are designed for billing and reimbursement rather than research purposes. The claims may have contained incomplete information or undetected coding errors or omissions. Information on sociodemographic factors such as educational background, income, etc. that can be used for matching were not available in this administrative claims database. The ability to determine the severity of diabetes or HBV infection was limited by the information contained in the claims. Furthermore, some patients who were placed in the diabetes-only group may have had undiagnosed HBV infection. In cases where a visit to the ED resulted in hospitalization, that incident was counted as an inpatient hospitalization and not an ED visit, which may have under-represented the number of ED visits in this patient population. While the population was geographically diverse, all patients were members of a large commercial health plan. The results may not be generalizable to patients with other types of insurance or to those who are uninsured.

As this analysis illustrates, the financial burden associated with diabetes and HBV infection, particularly in the presence of late-stage liver disease, can be considerable. It provides evidence that there is a potential to reduce the economic impact of HBV by vaccinating patients with diabetes following their diagnosis. Typically, health plans reimburse hepatitis B vaccination for patients with diabetes if delivered by the physician. Healthcare quality organizations could also potentially have a role in improving vaccination coverage. For example, the National Quality Forum (NQF) recognized hepatitis B vaccination in diabetes as one of the gaps in adult immunization measures and measure development. Development of hepatitis B vaccination measure in diabetes and endorsement of such measure by NQF may raise the significance of delivery of hepatitis B vaccination in patients with diabetes.

HBV infection increased the financial burden of patients with diabetes, particularly in patients with late-stage liver disease. Healthcare utilization and costs were higher among patients with both diabetes and HBV infection than in those with diabetes alone. These results suggest providers should consider vaccination against HBV infection among patients with diabetes who have not previously been vaccinated or infected with HBV.

## Materials and methods

### Data source and patient identification

This retrospective, observational analysis used data contained in the HealthCore Integrated Research Database (HIRD^SM^). The HIRD^SM^ contains medical and pharmacy claims data from 14 commercial health plans across the US. This claims analysis was conducted in compliance with state and federal laws, including the Health Insurance Portability and Accountability Act of 1996. As all claims data were from a limited dataset with de-identified patient information and no patients were identified, Institutional Review Board approval was not required.

Patients eligible for inclusion had at least one medical or pharmacy claim for diabetes (either type 1 or type 2) between January 1, 2006 and March 31, 2014 (the study period). Claims for HBV must have occurred during the intake period (between January 1, 2007 and March 31, 2013) to allow for 12-month pre- and post-index periods. The pre-index period was used to capture baseline characteristics. All patients were required to have 2 or more medical claims any time from January 2006 to March 2014 (at least 30 d apart) with an *International Classification of Diseases, Ninth Revision, Clinical Modification* (ICD-9-CM) diagnosis code (250.xx) suggesting diabetes or at least 1 medical claim with a diagnosis code for diabetes along with at least 1 pharmacy claim for a diabetes medication during the study period. Patients with a diagnosis code indicating the presence of hepatitis C (ICD-9-CM codes 070.44, 070.54, 070.70, 070.71, 070.41, 070.51, or V02.62) were excluded from the study to ensure utilization and cost results were attributable only to HBV infection.

Patients were then divided into one of 2 cohorts: the diabetes plus HBV infection cohort (cases) composed of diabetes patients who had 2 or more medical claims (at least 30 d apart) with diagnosis codes for HBV infection (ICD-9-CM code 070.2x or 070.3x); the diabetes-only cohort (controls) contained patients who had claims for diabetes during the intake period with no diagnosis codes for HBV infection at any point during the study period. The index date for cases was defined as the date of the first medical claim for HBV. The index date for controls was the date of the first medical or pharmacy claim for diabetes in the diabetes-only cohort observed after 12 months from the start of eligibility; this was to ensure all patients had at least 12 months of pre-index health plan eligibility. Patients were followed until they disenrolled or end of study period (March 31, 2014).

### Propensity score matching

Propensity score matching was used to adjust for measured confounders between study cohorts.^19^ Logistic regression propensity scores used observed patient demographics (eg, age, gender, US region, etc.) and baseline clinical characteristics (eg, comorbidities and use of healthcare resources not related to HBV infection). The logistic regression analysis weighed the predictor variables that best discriminated between the two groups. This formula was applied to each patient's values on all predictor variables to produce a predicted score, which was that patient's propensity score. Variables included in the final propensity score model (Appendix) were selected based on previous literature establishing their biologic rationale and confirmed by the balance achieved between cohorts after matching on propensity scores. Patients with diabetes plus HBV infection were matched with patients with diabetes only based on the eighth digit of the propensity score using a 1:4 greedy matching algorithm.^20,21^

### Disease severity

Adapted Diabetes Comorbidity Severity Index (aDCSI) was used in propensity score matching to adjust for severity of diabetes. Based on the presence of diabetes-related comorbidities, aDCSI produces scores of 0 (no abnormality), 1 (some abnormality), or 2 (severe abnormality) in 7 complication categories: retinopathy, nephropathy, neuropathy (which has only 2 levels: 0=not present; 1=abnormal), cerebrovascular complications, cardiovascular complications, peripheral vascular disease, and metabolic complications.^22,23^ The total combined score may range from 0 to 13. For the purposes of this analysis and based on expert clinical opinion, an aDCSI score of 0 designated mild diabetes; 1 to 4 designated moderate diabetes; and a score of 5 to 13 designated severe diabetes.

Late-stage liver disease was identified based on the presence of ICD-9-CM diagnostic codes during the follow-up period associated with liver disease and were assigned to mutually exclusive categories in descending priority beginning with liver transplant, fulminant hepatic failure, liver cancer, decompensated cirrhosis, and cirrhosis.^24^ That is, if a patient had 2 of these conditions, the patient was assigned to the condition higher in hierarchy, indicating more severe disease.

### Outcome measures

Healthcare utilization and costs were assessed for inpatient hospitalizations; emergency department (ED) visits; office visits (all-cause, gastroenterologist, and infectious disease specialist); outpatient services (such as laboratory procedures); skilled nursing facility services; and pharmacy prescriptions. All-cause costs were calculated as plan-paid and patient-paid costs, which included all coinsurance, deductible, and co-payments. Costs were adjusted to 2014 dollars based on the Consumer Price Index^25^ and were annualized to account for different follow-up times among patients. Total medical costs were a sum of inpatient, ED, office visit, outpatient costs, and skilled nursing facility costs; total costs included both total medical plus pharmacy costs.

### Statistical analysis

Descriptive statistics, such as means (standard deviation [SD]) and relative frequencies, were reported for continuous and categorical data, respectively. Patient characteristics, which were obtained from health plan enrollment data in HIRD^SM^, were compared statistically between the two groups using the diabetes-only group as the reference group. The χ^2^ test was used for dichotomous variables and *t*-test was used for continuous dependent variables. The χ^2^ test and *t*-test were used only for pre-index demographic and clinical characteristics. Statistical significance was set at *p*<0.05.

Incremental healthcare utilization and between-group differences in costs were calculated using multivariate models controlling for baseline insulin use and use of antidiabetic agents associated with hepatotoxicity (ie, sulfonylureas, α-glucosidase inhibitors, biguanides, and thiazolidinediones).^26^ The negative binomial regression with log-link function was used to analyze healthcare utilization; between-group cost differences were analyzed using generalized linear models with a gamma distribution and log-link function. Estimated β coefficients obtained by the generalized linear models were exponentiated to calculate the incremental differences between groups. The distribution of incremental costs were converted to actual cost (in dollars) to provide meaningful results for interpretation.

## Appendix. Variables Included in Propensity Score Model

• Age on index date
• Gender
• Geographic region on index date
• Index year
• Length of pre-index eligibility
• Presence of diabetes during pre-index period
• aDCSI score
• Comorbidities^a^: cerebrovascular disease, chronic obstructive pulmonary disease, congestive heart failure, coronary artery disease, dementia, hemiplegia or paraplegia, HIV/AIDS, hypertension, hyperlipidemia, malignancy, metastatic solid tumor, moderate or severe renal disease, other liver disease, peptic ulcer disease, peripheral vascular disease, rheumatological disease
• Frequency of office visits *not* related to HBV or its related complications
• Pre-index hospitalization *not* related to HBV or its related complications

aDCSI= adapted Diabetes Comorbidity Severity Index; HBV=hepatitis B virus; HIV=human immunodeficiency virus

^a^Comorbidities identified in the pre-index period based on the presence of *International Classification of Diseases, Ninth Revision, Clinical Modification* diagnosis codes
